# What is the impact of SARS-CoV-2 pandemic on antimicrobial stewardship programs (ASPs)? The results of a survey among a regional network of infectious disease centres

**DOI:** 10.1186/s13756-022-01152-5

**Published:** 2022-08-29

**Authors:** Agnese Comelli, Camilla Genovese, Andrea Lombardi, Chiara Bobbio, Luigia Scudeller, Umberto Restelli, Antonio Muscatello, Spinello Antinori, Paolo Bonfanti, Salvatore Casari, Antonella Castagna, Francesco Castelli, Antonella d’Arminio Monforte, Fabio Franzetti, Paolo Grossi, Matteo Lupi, Paola Morelli, Stefania Piconi, Massimo Puoti, Luigi Pusterla, Angelo Regazzetti, Marco Rizzi, Stefano Rusconi, Valentina Zuccaro, Andrea Gori, Alessandra Bandera, the ASP Lomb Study Group, Andrea Giacomelli, Marianna Rossi, Raffaele Bruno, Silvia Garilli, Ripa Marco, Liana Signorini, Francesca Bai, Angelo Pan, Marco Merli, Davide Ricaboni, Chiara Molteni, Simone Vasilij Benatti, Barbara Castiglioni, Cristina Rovelli, Manuela Piazza, Marco Franzetti

**Affiliations:** 1 Infectious Diseases Unit, Foundation IRCCS Ca’ Granda Ospedale Maggiore Policlinico, Via Francesco Sforza 35, 20122 Milan, Italy; 2 University of Milan, Milan, Italy; 3 Research and Innovation Unit, IRCCS Azienda Ospedaliero-Universitaria di Bologna, Bologna, Italy; 4 LIUC Cattaneo University, Castellanza, VA Italy; 5 Department of Biomedical and Clinical Sciences DIBIC, Luigi Sacco, Università Degli Studi di Milano, Milan, Italy; 6 Infectious Diseases Unit, San Gerardo Hospital, University of Milano-Bicocca, Monza, Italy; 7 Unit of Infectious Diseases, Carlo Poma Hospital, ASST Mantova, Mantua, Italy; 8 Infectious Diseases Unit, IRCCS San Raffaele Scientific Institute, Milan, Italy; 9 University Department of Infectious and Tropical Diseases, University of Brescia and ASST Spedali Civili, Brescia, Italy; 10 Department of Health Sciences, Clinic of Infectious Diseases, ASST Santi Paolo E Carlo, University of Milan, Milan, Italy; 11 Infectious Diseases Unit, ASST Valle Olona, Busto Arsizio Hospital, Busto Arsizio, Italy; 12 Infectious and Tropical Diseases Unit, Department of Medicine and Surgery, University of Insubria and ASST-Sette Laghi, Varese, Italy; 13 Division of Infectious Diseases, ASST Cremona, Cremona, Italy; 14 Infectious Disease Unit, IRCCS Humanitas Research Hospital, Rozzano, Italy; 15 Infectious Diseases Unit, Alessandro Manzoni Hospital, ASST Lecco, Lecco, Italy; 16 Infectious Diseases, Hospital Niguarda, Milan, Italy; 17 Division of Infectious Diseases, ASST Lariana, Como, Italy; 18 UOC Di Malattie Infettive E Tropicali - Ospedale Delmati di Sant’Angelo Lodigiano, ASST Di Lodi, Lodi, Italy; 19 Infectious Diseases Unit, ASST Papa Giovanni XXIII, Bergamo, Italy; 20 Infectious Diseases Unit, Ospedale Civile di Legnano, ASST Ovest Milanese, Legnano, Italy; 21 Division of Infectious Diseases I, Fondazione IRCCS Policlinico San Matteo, Pavia, Italy

**Keywords:** COVID-19, SARS-CoV-2, Antimicrobial stewardship, Multidrug resistant organisms, Antimicrobials use

## Abstract

Discontinuation of antimicrobial stewardship programs (ASPs) and increased antibiotic use were described during SARS-CoV-2 pandemic. In order to measure COVID-19 impact on ASPs in a setting of high multidrug resistance organisms (MDRO) prevalence, a qualitative survey was designed. In July 2021, eighteen ID Units were asked to answer a questionnaire about their hospital characteristics, ASPs implementation status before the pandemic and impact of SARS-CoV-2 pandemic on ASPs after the 1st and 2nd pandemic waves in Italy. Nine ID centres (50%) reported a reduction of ASPs and in 7 cases (38.9%) these were suspended. After the early pandemic waves, the proportion of centres that restarted their ASPs was higher among the ID centres where antimicrobial stewardship was formally identified as a priority objective (9/11, 82%, vs 2/7, 28%). SARS-CoV-2 pandemic had a severe impact in ASPs in a region highly affected by COVID-19 and antimicrobial resistance but weaknesses related to the pre-existent ASPs might have played a role.

## Background

Since its first identification in late 2019, the novel acute respiratory syndrome coronavirus (SARS-CoV-2) has attracted the attention of healthcare workers and researchers worldwide.

Its impact on antimicrobial resistance (AMR) has also become a matter of discussion. Studies conducted over the last two years have shown that antibiotics are largely prescribed to patients with coronavirus disease (COVID-19), even though bacterial co-infections are infrequent so far [1, 2].

Antimicrobial stewardship programs (ASPs) aim to curb the growing threat of antibiotic resistance by optimizing the use of antibiotics. However, most established ASPs have been discontinued and resources and staff have been reallocated to contribute to COVID-19 pandemic response [3]. In a survey conducted in the UK, more than 60% of participating centres reported that COVID-19 had a negative impact on routine AMS activities with a decrease in stewardship rounds, multidisciplinary meetings and point prevalent surveys [4].

Although it might be too early to estimate the consequences of the pandemic on AMR progression, early studies showed that the interruption of ASPs led to an overall increase in broad spectrum antibiotics use and multidrug resistant (MDR) healthcare-associated infections (HAIs) incidence [3, 5]. Conversely, in settings where strong ASPs were maintained, antibiotic consumption decreased during the pandemic [6].

Italy, and in particular the Lombardy Region, has been among the areas with the highest number of SARS-CoV-2 cases since the beginning of the pandemic [7]. Moreover, Italy is one of the European countries with the highest prevalence of MDRO [8]. Therefore, assessing the impact of the COVID-19 pandemic on ASPs among Lombardy hospitals is particularly relevant in order to estimate the pandemic long-term consequences in a setting in which AMR prevalence is already high.

Here, we present the results of a survey designed to investigate ASPs status before, during and after the SARS-CoV-2 pandemic.

## Methods

### Study design and population

A qualitative survey using a 59-item questionnaire was developed by a group of experts in AMS from Fondazione IRCSS Ca’ Granda Ospedale Maggiore Policlinico di Milano, Lombardy region, northern Italy.

The questionnaire was hosted in REDCap data capture tool and the 18 Infectious Disease Units belonging to the Lombardy ID Network were invited to complete the survey.

An ID physician from the AMS team was asked to complete the survey.

The questionnaire was uploaded on the 27th July 2021 and the addressees were asked to answer within the 5th August 2021.

The survey included 3 sections: (1) characteristics of ﻿the hospitals including: name and type of hospital (teaching, private, public), number of hospital beds and presence of key wards in terms of AMS activity (e.g., ICU, HSCT and solid transplant unit); (2) ASPs implementation status before the pandemic and (3) impact of SARS-CoV-2 pandemic on ASPs.

Section 2 of the survey was inspired by the position paper published in 2018 by *Pulcini* et al. where the authors identified 7 core elements to develop, evaluate and audit an ASP [9].

Section 3 included 11 questions about ASPs changes during and after the first and the second wave of SARS-CoV-2 pandemic in Italy (1st wave: Feb 2020-Jun 2020; 2nd wave: Sept 2020-Dec 2020). Management of health care professionals, ﻿antibiotic consumption and antimicrobial resistance (AMR) were investigated.

### Statistics

Continuous variables were summarized as mean and standard deviation, and categorical variables as absolute and relative frequencies.

## Results

All the 18 ID Units of the Lombardy ID Network answered the survey. Nearly 90% of the responding centres were public hospitals (88.9%) and half of the ID units were affiliated to the local Universities.

Almost 50% of the hospitals had 500–750 beds and around 45% more than 1000. ICUs are present in all but one hospital whereas 60% and 39% of them have HSCT and transplantation units respectively (see Table 1 for more details).

**Table 1:** Descriptive analysis of the survey’s answers by the participating ID units (*n* = 18)

	Values
Type of hospital, *n* (%)	
Private	2 (11.1)
Public	16 (88.9)
Teaching hospital, *n* (%)	9 (50%)
Number of beds, *n* (%)	
< 500	1 (5.5)
500–750	9 (50)
750–1000	5 (27.8)
> 1000	3 (16.7)
Presence of the following wards, *n* (%)	
ICU	17 (94.4)
Transplantation unit	7 (38.9)
HSCT	11 (61.1)
Presence of molecular identification of CR, *n* (%)	15 (83.3)
AMS formally identified as a priority objective by the hospital management, *n* (%)	11 (61.1)
AMS formally implemented before SARS-CoV-2 pandemic, *n* (%)	13 (72.2)
AMS has been implemented since, *n* (%)	
< 6 months	3 (23.1) ^a^
> 6 months–< 12 months	1 (7.7) ^a^
> 12 months–< 24 months	1 (7.7) ^a^
> 24 months	8 (61.5) ^a^
Sufficient financial support AMS activities, *n* (%)	1 (7.7) ^a^
Staffing standards^b^ for AMS activities fulfilled, *n* (%)	5 (38.5) ^a^
Formal/written ASP/strategy, *n* (%)	9 (69.2) ^a^
Healthcare professional identified as a leader for AMS activities, *n* (%)	8 (61.5) ^a^
Formal/written definition of roles and responsibilities of AMS team members, *n* (%)	3 (23.1) ^a^
Regular report on antimicrobial use/prescription trend, *n* (%)	5 (38.5) ^a^
Educational resources to support antimicrobial use, *n* (%)	14 (77.8)
Regular training of AMS team members, *n* (%)	5 (38.5) ^a^
Multidisciplinary AMS team^c^, *n* (%)	10 (76.9) ^a^
Adequate technology services for AMS, *n* (%)	3 (23.1) ^a^
Antimicrobial formulary for unrestricted, restricted or permitted antibiotics, *n* (%)	16 (88.9)
AMS team review/audit of therapy courses for specified antimicrobial agents or clinical conditions, *n* (%)	5 (38.5) ^a^
Regular monitoring of quality of antimicrobial use at the unit and/or hospital wide level, *n* (%)	8 (44.4)
Regular monitoring of quantity of antimicrobial use at the unit and/or hospital wide level, *n* (%)	16 (88.9)
Monitoring of compliance with one or more of the specific interventions of AMS, *n* (%)	2 (15.4) ^a^
Monitoring of antibiotic susceptibility rates for a range of key bacteria, *n* (%)	15 (83.3)
Methicillin resistant *Staphylococcus aureus* (MRSA)	15 (83.3)
Carbapenem-Resistant *Enterobacterales* (CRE)	15 (83.3)
*Escherichia coli* ESBL +	15 (83.3)
Vancomycin resistant *enterococci* (VRE)	15 (83.3)
*MDR A. baumanni*	14 (77.8)
*MDR P. aeruginosa*	15 (83.3)
*Candida* spp	6 (33.3)
Sharing of hospital-specific reports with prescribers, *n* (%)	
On the quantity of antimicrobials prescribed/dispensed/purchased	7 (38.9)
On antibiotic susceptibility rates	8 (44.4)

*AMS* Antimicrobial stewardship, *ASP* Antimicrobial stewardship program, *MDRO* Multidrug resistant organisms

^a^The percentage calculated on the 13 ID centres where a formal AMS program has been implemented

^b^1 infection control nurse/300 beds; 1 AMS physician/1000 beds

^c^ID specialist + at least one member from Pharmacy, Microbiology, IPC and other specialties physicians

Before the SARS-CoV-2 pandemic, 13 out of 18 (72.2%) ID Units had implemented a structured and formally approved ASP and, among them, 8 ASPs (61.5%) have been implemented since more than 24 months.

All but one centre reported to receive insufficient financial support for AMS activities and AMS was formally identified as a priority objective by the hospital management in 11/18 (61%) participating hospitals.

Ten out of the 13 structured and formally approved ASPs (76.9%) were equipped by a multidisciplinary team that included at least one ID specialist, a clinical microbiologist or a lab microbiologist, a member of the infection prevention and control (IPC) unit and a pharmacist.

However, the remaining centres without a formally approved ASP were able to implement some AMS-related activities.

Specifically, educational resources to support antimicrobial use were promoted in 77.8% of ID centres, about 90% hospitals shared an antimicrobial formulary for unrestricted, restricted or permitted antibiotics, 44.4% and 88.9% regularly monitored quality and quantity of consumed antimicrobials, respectively. Moreover, 83.3% of ID units regularly monitored antibiotic susceptibility rates for a range of key bacteria and 8 units (44.4%) shared those reports with prescribers. See Table 1 for further data.

During the first 2 pandemic waves (1st wave: Feb 2020-Jun 2020; 2nd wave: Sept 2020-Dec 2020) all the ID units were converted to COVID-19 wards and in all cases the AMS team members were shifted to clinical activity related to COVID-19.

**Fig. 1:**
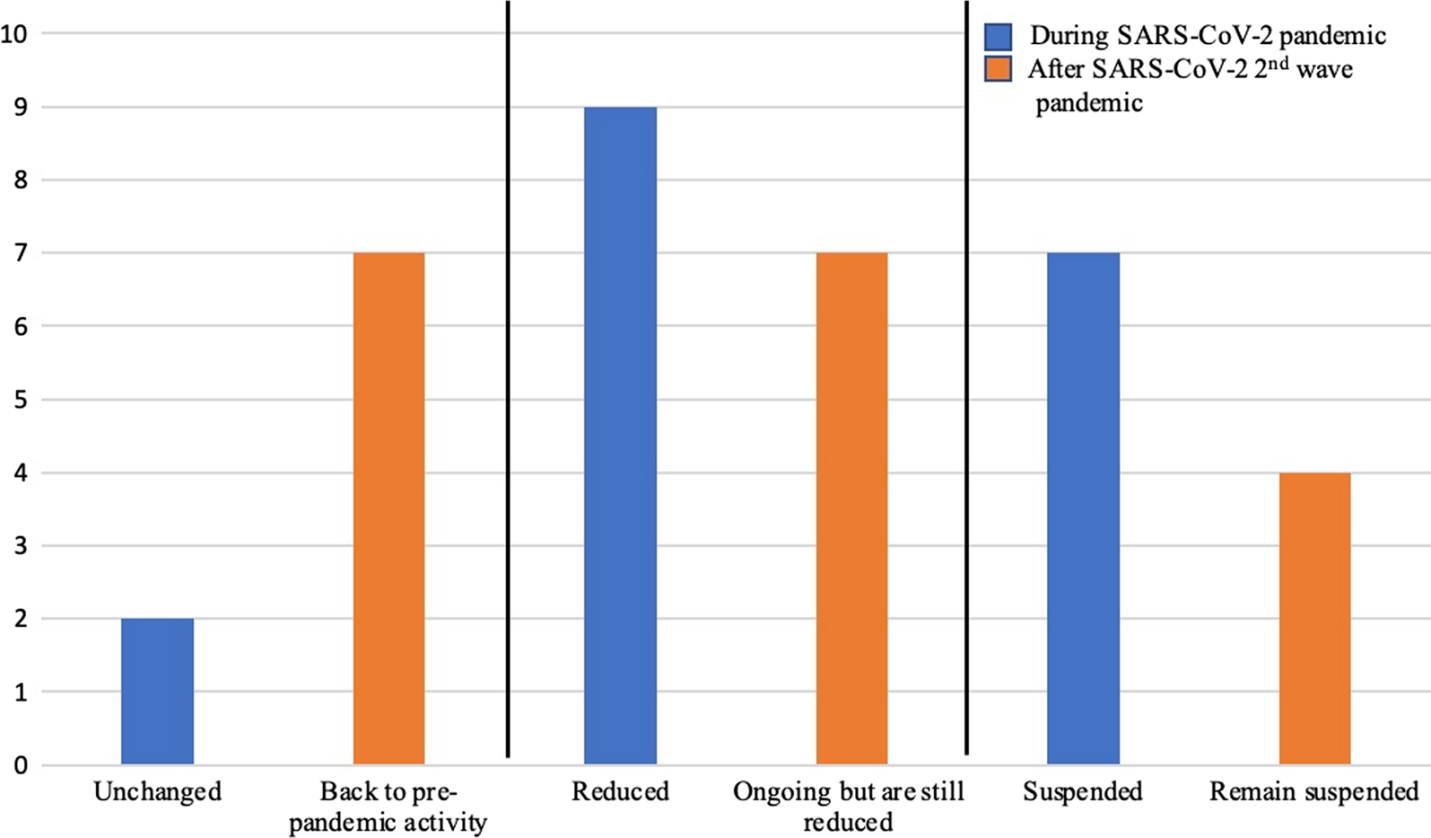
ASPs changes during and after the first 2 waves of SARS-CoV-2 pandemic

As a consequence, a reduction of ASPs was registered in 9 ID units (50%) and a complete suspension was reported in 7 cases (38.9%). Two ID units (11.1%), both of which with a structured and formally approved ASP before the pandemic, were able to keep their projects running (Fig. 1).

The proportion of centres with formal monitoring of antimicrobial use/prescription remained stable before and after the first 2 waves of the pandemic (38.5% versus 33% respectively).

During the pandemic, the ratio of ID units that maintained (unchanged or reduced) ASPs differed among those centres for which hospital management formally identified ASPs as a priority objective as compared to the ID units that did not identify AMS as a primary target (9/11, 82% versus 2/7, 28%).

Similarly, the proportion of centres which maintained (unchanged or reduced) ASPs was higher between the ID units that promoted educational resources to support antimicrobial use as compared to those that did not promote educational activities (9/14, 63% versus 2/4, 50%).

HAI outbreaks sustained by multidrug resistant organisms (MDRO) were observed in 89% of participating hospitals during COVID-19 pandemic, being vancomycin-resistant enterococci (VRE), MDR *Acinetobacter baumanii* and MDR *Pseudomonas aeruginosa* the most frequently involved pathogens. No Candida auris cases were registered (Table 2).

**Table 2:** SARS-CoV-2 pandemic impact on ASPs

	Values
ASPs during SARS-CoV-2 pandemic, *n* (%)	
Unchanged	2 (11.1)
Reduced	9 (50)
Suspended	7 (38.9)
ASPs post SARS-CoV-2 pandemic, *n* (%)	
Back to pre-pandemic activity	7 (38.9)
Ongoing but are still reduced	7 (38.9)
Remain suspended	4 (22.2)
Monitoring of high-cost antibiotic prescription, *n* (%)	6 (33.3)
Regular reports on antimicrobial usage, *n* (%)	6 (33.3)
Regular reports on the epidemiology of microbial isolates, *n* (%)	9 (50)
Ordinary wards converted into COVID-19 wards, *n* (%)	18 (100)
Number of beds dedicated to COVID-19 patients (April 2020)	
50	3 (16.7)
50–100	10 (55.6)
100–250	5 (27.8)
250–500	0
> 500	0
Number of beds dedicated to COVID-19 patients (August 2020)	
50	16 (88.9)
50–100	1 (5.6)
100–250	1 (5.6)
250–500	0
> 500	0
AMS team members shifted to clinical activity in COVID-19 wards, *n* (%)	13 (100) ^a^
MDRO hospital acquired infections outbreaks in COVID-19 wards during the pandemic, *n* (%)	16 (88.9)
Carbapenem-resistant *Enterobacterales* (CRE)	8 (44.4)
Vancomycin-resistant enterococci (VRE)	11 (61.1)
*MDR A. baumanni*	11 (61.1)
*MDR P. aeruginosa*	11 (61.1)
*Candida auris*	0

*AMS* Antimicrobial stewardship, *ASP* Antimicrobial stewardship program, *MDRO* Multidrug resistant organisms

^a^The percentage calculated on the 13 ID centres where a formal AMS program has been implemented

After the 2 pandemic waves, the proportion of centres that restarted their ASPs at the pre-pandemic level or continued them even if reduced was higher among the ID units where AMS was formally identified as a priority objective by the hospital management (9/11, 82%, versus 2/7, 28%).

Likewise, a higher percentage of centres restarted their ASPs at the pre-pandemic level or continued them even if reduced among the ID units where educational resources to support antimicrobial use were promoted as compared to those that did not (9/14, 64.3%, versus 2/4, 50%).

## Discussion

SARS-CoV-2 pandemic heavily impacted ASPs of a regional network of 18 Italian ID units with a reduction or a temporarily suspension of previously ongoing ASPs in nearly 90% of included hospitals.

To our knowledge this is the first report conducted in a setting of high prevalence of MDROs. A recent, large survey conducted among 95 hospitals in the UK showed a ﻿negative impact of COVID-19 on AMS activity in 65% of included hospitals [4] but our figures are definitely worse.

Our survey also pointed out that after the first 2 pandemic waves, less than 50% of the ID units were able to restore their pre-pandemic ASPs even if some improvements in the recovery of AMS activities were achieved between the 2 waves and immediately after.

Multiple reasons explain the negative impact of COVID-19 on AMS activities. First, the fight against SARS-CoV-2 absorbed most of the economic resources of the health care system and, consequently, ASPs, as well as other non-primary health services, were inevitably sacrificed. Moreover, other factors related to COVID-19 management were potentially linked to disruption of antimicrobial surveillance and consequent increased use of antimicrobials and MDRO outbreaks, such as ﻿the focus of healthcare workers on self-protection, hospital overcrowding, ﻿low health care workers (HCW):patient ratio, ﻿personal protective equipment shortage etc [4, 5, 10].

However, in addition to the COVID-19 impact, the survey results highlighted some weaknesses of the pre-existent ASPs that might have played a role in the discontinuation of these projects during the pandemic and in their late restart after the first 2 waves.

Indeed, before the pandemic, only few hospitals dedicated sufficient economic and personnel resources to AMS activities and, despite the endemicity of MDROs in Italy, almost 40% of the participating ID centres have only recently started the AMS programme.

Although the small number of ID Units included did not allow us to identify any statistically significant correlation, the absence of a strong commitment from hospital management may have influenced the discontinuation of ASPs and hindered their return to pre-pandemic status.

In a ﻿tertiary hospital in Singapore, the hospital management worked in advance with the AMS team to be prepared for the pandemic in terms of antibiotic supply and prescription recommendation. As a consequence, in this setting, there was ﻿no increase in antimicrobial prescribing and no significant difference in antimicrobial prescribing quality indicators during the COVID-19 pandemic [11].

Finally, nearly 90% of the participating ID units reported MDRO outbreaks in COVID-19 wards during the pandemic. This data was not compared with other wards or with pre-pandemic phases. Outbreaks sustained by Carbapenem-Resistant Enterobacterales (CRE), Vancomycin Resistant enterococci (VRE), MDR *P. aeruginosa* and MDR *A. baumanni* have also been reported in countries with lower endemicity for MDR pathogens [12–15]. These data are probably the most direct markers of the effect of discontinuation of IPC and ASPs.

Our study has some limitations. First, as with all qualitative studies, the lack of quantitative data hinders an accurate measurement of the COVID-19 impact at different stages of the pandemic. Second, the small sample size allowed only a descriptive analysis.

In contrast, one of the strengths of this study is the inclusion of all the ID units operating in a large Italian region (with a resident population of almost 10 million subjects) that is both highly endemic for MDR infection and has been particularly affected by SARS-CoV-2 pandemic.

In addition, we employed a comprehensive and structured evaluation of ASPs that could be used to reassess the long-term improvements in each ID unit.

## Conclusions

The SARS-CoV-2 pandemic had a great impact on ASPs in Lombardy Region but some weaknesses were evident even before the health emergency.

In addressing the COVID-19 pandemic or the next public health emergency, we cannot forget another long-standing pandemic, antimicrobial resistance. Sufficient economic and public health resources must be allocated to lay the foundation for a stable, structured and efficient AMS system.

Furthermore, networking ID units that share similar epidemiology and similar difficulties could be a first step in building a coordinated local monitoring and feedback system.

## Data Availability

All data generated or analysed during this study are included in this published article.
